# Supplementary material for: Regulation of trehalase activity by multi-site phosphorylation and 14-3-3 interaction
Source: Sci Rep. 2021 Jan 13;11:962. doi: 10.1038/s41598-020-80357-3 (PMC7806596; doi:10.1038/s41598-020-80357-3)

Supplementary Information for:

Regulation of trehalase activity by multi-site phosphorylation and 14-3-3 interaction

Lisa Dengler[1], Mihkel Örd[2], Lucca M. Schwab[1], Mart Loog[2] and Jennifer C. Ewald[1,*]

[1] University of Tübingen, Interfaculty Institute of Cell Biology, 72076 Tübingen, Germany.

[2] University of Tartu, Institute of Technology, Tartu 50411, Estonia.

*Correspondence: jennifer.ewald@ifiz.uni-tuebingen.de

Table S1: Strains used in this study.

Table S2: Plasmids used in this study.

Table S3: Absolute trehalose concentrations.

Figure S1: Further characterization of the Nth1 reporter construct.

Figure S2: Control experiments related to Nth1 activity.

Figure S3: S20 and S21 are phosphorylated *in vitro* and *in vivo* but not shielded by Bmh1.

Figure S4: Further characterization of the N-terminal region surrounding S20 and S21.

Supplementary References

Additional Supplementary Information: Images of all uncropped gels and blots

Supplementary Table S1, related to experimental procedures: Strains used in this study.

name	genotype (all strains are prototrophic and based on *S. cerevisiae* W303)	comment/source
KK001b	*MAT a, ADE, LEU, HIS, TRP, nth1::kanMX, URA*	deletion of *nth1*
LD001 (full-length)	*MAT a, ADE, LEU, HIS, TRP, nth1::kanMX, nth1pr::NTH1pr-NTH1^{WT}-URA3*	after deletion of *nth1*, a pRS406 plasmid (Stratagene) containing *NTH1* promoter-*NTH1* was integrated into the genomic *NTH1* promoter
LD002 (full-length)	*MAT a, ADE, LEU, HIS, TRP, nth1::kanMX, nth1pr::NTH1pr-NTH1^{S66A}-URA3*	like above, but *NTH1^{S66A}*
LD003 (full-length)	*MAT a, ADE, LEU, HIS, TRP, nth1::kanMX, nth1pr::NTH1pr-NTH1^{S20,21,60,83A} -URA3*	like above, but *NTH1^{S20,21,60,83A}*
LD004 (full-length)	*MAT a, ADE, LEU, HIS, TRP, nth1::kanMX, nth1pr::NTH1pr-NTH1^{S20,21A}-URA3*	like above, but *NTH1^{S20,S21A}*
LD018 (full-length)	*MAT a, ADE, LEU, HIS, TRP, nth1::kanMX, nth1pr::NTH1pr-NTH1^{S20A}-URA3*	like above, but *NTH1^{S20A}*
LD019 (full-length)	*MAT a, ADE, LEU, HIS, TRP, nth1::kanMX, nth1pr::NTH1pr-NTH1^{S21A}-URA3*	like above, but *NTH1^{S21A}*
LD015 (full-length)	*MAT a, ADE, LEU, HIS, TRP, nth1::kanMX, nth1pr::NTH1pr-NTH1^{L19A}-URA3*	like above, but *NTH1^{L19A}*
LD009 (full-length)	*MAT a, ADE, LEU, HIS, TRP, nth1::kanMX, nth1pr::NTH1pr-NTH1^{S60,83A}-URA3*	like above, but *NTH1^{S60,83A}*
LD046 (full-length)	*MAT a, ADE, LEU, HIS, TRP, nth1::kanMX, nth1pr::NTH1pr-NTH1^{S60A}-URA3*	like above, but *NTH1^{S60A}*
LD047 (full-length)	*MAT a, ADE, LEU, HIS, TRP, nth1::kanMX, nth1pr::NTH1pr-NTH1^{S83A}-URA3*	like above, but *NTH1^{S83A}*
KK040 (full-length)	*MAT a, ADE, LEU, HIS, TRP, nth1::kanMX, nth1pr::NTH1pr-NTH1-truncated-URA3*	like above, but *NTH1$^{\Delta 1\text{-}24}$*
KK059 (full-length)	*MAT a, ADE, LEU, HIS, TRP, nth1::kanMX, nth1pr::NTH1pr-NTH1^{S20,21E}-URA3*	like above, but *NTH1^{S20,S21E}*
LD014 (full-length-FLAG)	*MAT a, ADE, LEU, HIS, TRP, nth1::kanMX, nth1pr::NTH1pr-NTH1-3XFLAG -URA3*	like above, but *NTH1^{WT}*-3XFLAG
JE107 (reporter)	*MAT a, ADE, LEU, HIS, TRP, ura3::URA3 NTH1^{WT} -reporter-3XFLAG*	a pRS406 plasmid (Stratagene) containing *NTH1* promoter-*NTH1^{WT}*-reporter-3XFLAG (first 95 aa) was integrated into the *URA3* locus
LD005 (reporter)	*MAT a, ADE, LEU, HIS, TRP, ura3::URA3 NTH1^{S66A} -reporter-3XFLAG*	like above, but *NTH1^{S66A}*-reporter-3XFLAG
LD013 (reporter)	*MAT a, ADE, LEU, HIS, TRP, ura3::URA3 NTH1^{S20,21,60,83A}-reporter-3XFLAG*	like above, but *NTH1^{S20,21,60,83A}*-reporter-3XFLAG
LD007 (reporter)	*MAT a, ADE, LEU, HIS, TRP, ura3::URA3 NTH1^{S20,21A} -reporter-3XFLAG*	like above, but *NTH1^{S20,21A}*-reporter-3XFLAG

LD021 (reporter)	*MAT a, ADE, LEU, HIS, TRP, ura3::URA3 NTH1^{S20A} -reporter-3XFLAG*	like above, but *NTH1*^{S20A}-reporter-3XFLAG
LD022 (reporter)	*MAT a, ADE, LEU, HIS, TRP, ura3::URA3 NTH1^{S21A} -reporter-3XFLAG*	like above, but *NTH1*^{S21A}-reporter-3XFLAG
LD008 (reporter)	*MAT a, ADE, LEU, HIS, TRP, ura3::URA3 NTH1^{S60,83A}-reporter-3XFLAG*	like above, but *NTH1*^{S60,83A}-reporter-3XFLAG
LD011 (reporter)	*MAT a, ADE, LEU, HIS, TRP, ura3::URA3 NTH1^{S60A}-reporter-3XFLAG*	like above, but *NTH1*^{S60A}-reporter-3XFLAG
LD012 (reporter)	*MAT a, ADE, LEU, HIS, TRP, ura3::URA3 NTH1^{S83A}-reporter-3XFLAG*	like above, but *NTH1*^{S83A}-reporter-3XFLAG
LD010 (reporter)	*MAT a, ADE, LEU, HIS, TRP, ura3::URA3 NTH1^{L19A}-reporter-3XFLAG*	like above, but *NTH1*^{L19A}-reporter-3XFLAG
LD023 (reporter)	*MAT a, ADE, LEU, HIS, TRP, ura3::URA3 NTH1^{E24A}-reporter-3XFLAG*	like above, but *NTH1*^{E24A}-reporter-3XFLAG
KK018 (reporter)	*MAT a, ADE, LEU, HIS, TRP, ura3::URA3 NTH1^{S21,60,66,83A}-reporter-3XFLAG*	like above, but *NTH1*^{S21,60,66,83A} -reporter-3XFLAG
KK019 (reporter)	*MAT a, ADE, LEU, HIS, TRP, ura3::URA3 NTH1^{S20,60,66,83A}-reporter-3XFLAG*	like above, but *NTH1*^{S20,60,66,83A}-reporter-3XFLAG
KK020 (reporter)	*MAT a, ADE, LEU, HIS, TRP, ura3::URA3 NTH1^{S20,21,60,66A}-reporter-3XFLAG*	like above, but *NTH1*^{S20,21,60,66A}-reporter-3XFLAG
KK021 (reporter)	*MAT a, ADE, LEU, HIS, TRP, ura3::URA3 NTH1^{S60,66,83A}-reporter-3XFLAG*	like above, but *NTH1* ^{S60,66,83A}-reporter-3XFLAG
KK022 (reporter)	*MAT a, ADE, LEU, HIS, TRP, ura3::URA3 NTH1^{S20,21,66,83A}-reporter-3XFLAG*	like above, but *NTH1* $^{S20,21,66.83A}$-reporter-3XFLAG
KK037 (reporter)	*MAT a, ADE, LEU, HIS, TRP, ura3::URA3 NTH1-reporter-truncated-(25-95)-3XFLAG*	like above, but *NTH1$^{\Delta 1\text{-}24}$-reporter-3XFLAG*
LD048 (reporter)	*MAT a, ADE, LEU, HIS, TRP, ura3::URA3 NTH1^{S20,21E}-reporter-3XFLAG*	like above, but *NTH1*^{S20,21E}-reporter-3XFLAG
LD044 (reporter)	*MAT a, ADE, LEU, HIS, TRP, ura3::URA3 NTH1$^{\Delta 1\text{-}24,\ S66,83A}$ -reporter-truncated-3XFLAG*	like above, but *NTH1$^{\Delta 1\text{-}24,\ S66,83A}$ -reporter-3XFLAG*
LD030 (untagged control)	*MAT a, ADE, LEU, HIS, TRP, nth1::kanMX, nth1pr::NTH1pr-NTH1^{WT} -URA3, Bmh1::bmh1-V5- HphMX*	after deletion of *nth1*, *BMH1 was* 3PK-tagged at the endogenous locus by homologous recombination and a plasmid containing *NTH1* promoter- *NTH1*^{WT} was inserted into the genomic *NTH1* promoter
KK014	*MAT a, ADE, LEU, HIS, TRP, nth1::kanMX, nth1pr::NTH1pr-NTH1^{WT}-3XFLAG -URA3, Bmh1::bmh1-V5- HphMX*	*BMH1*-3PK tag was inserted in LD014
LD024	*MAT a, ADE, LEU, HIS, TRP, nth1::kanMX, nth1pr::NTH1pr-NTH1^{S66A}-3XFLAG -URA3, Bmh1::bmh1-V5- HphMX*	like above, but *NTH1*^{S66A}-3XFLAG
LD026	*MAT a, ADE, LEU, HIS, TRP, nth1::kanMX, nth1pr::NTH1pr-NTH1^{S20,21,60,66,83A}-3XFLAG -URA3, Bmh1::bmh1-V5- HphMX*	like above, but *NTH1*^{S20,21,60,66,83A}-3XFLAG
LD027	*MAT a, ADE, LEU, HIS, TRP, nth1::kanMX, nth1pr::NTH1pr-NTH1^{S60A}-3XFLAG -URA3, Bmh1::bmh1-V5- HphMX*	like above, but *NTH1*^{S60A}-3XFLAG

LD028	*MAT a, ADE, LEU, HIS, TRP, nth1::kanMX, nth1pr::NTH1pr-NTH1*^{S83A}*-3XFLAG -URA3, Bmh1::bmh1-V5- HphMX*	like above, but *NTH1*^{S83A}-3XFLAG
LD042	*MAT a, ADE, LEU, HIS, TRP, nth1::kanMX, nth1pr::NTH1pr-NTH1*^{L19A}*-3XFLAG -URA3, Bmh1::bmh1-V5- HphMX*	like above, but *NTH1*^{L19A}-3XFLAG
LD031	*MAT a, ADE, LEU, HIS, TRP, ura3::URA3 NTH1*^{WT} *-reporter-3XFLAG, Bmh1::bmh1-V5- HphMX*	like KK014, but in JE107
LD033	*MAT α, cln1Δ, cln2Δ, cln3::leu2, lexOPr-Cln1-Leu2, ADE2, his3::cyc1-Pr-lexO TF-his3, gph1::kanMX, TRP, nth1::natMX, nth1pr::NTH1pr-NTH1*^{WT}*-3XFLAG, Bmh1::bmh1-V5- HphMX*	Based on JE623b[1,2].<br>*BMH1 was* 3PK-tagged at the endogenous locus by homologous recombination, and a plasmid containing *NTH1* promoter-*NTH1*^{WT} was inserted into the genomic *NTH1* promoter
LD034	*MAT α, cln1Δ, cln2Δ, cln3::leu2, lexOPr-Cln1-Leu2, ADE2, his3::cyc1-Pr-lexO TF-his3, gph1::kanMX, TRP, nth1::natMX, nth1pr::NTH1pr-NTH1*^{S66A}*-3XFLAG, Bmh1::bmh1-V5- HphMX*	like above, but *NTH1*^{S66A}-3XFLAG
LD035	*MAT α, cln1Δ, cln2Δ, cln3::leu2, lexOPr-Cln1-Leu2, ADE2, his3::cyc1-Pr-lexO TF-his3, gph1::kanMX, TRP, nth1::natMX, nth1pr::NTH1pr-NTH1*^{S60A}*-3XFLAG, Bmh1::bmh1-V5- HphMX*	like above, but *NTH1*^{S60A}-3XFLAG
LD036	*MAT α, cln1Δ, cln2Δ, cln3::leu2, lexOPr-Cln1-Leu2, ADE2, his3::cyc1-Pr-lexO TF-his3, gph1::kanMX, TRP, nth1::natMX, nth1pr::NTH1pr-NTH1*^{S83A}*-3XFLAG, Bmh1::bmh1-V5-HphMX*	like above, but *NTH1*^{S83A}-3XFLAG
JE646-1 (reporter)	*MAT α, cln1Δ, cln2Δ, cln3::leu2, lexOPr-Cln1-Leu2, ADE2, his3::cyc1-Pr-lexO TF-his3, TRP, ura3::URA3 NTH1*^{WT} *-reporter-3XFLAG*	Based on JE611b [1,2].<br>a pRS406 plasmid (Stratagene) containing *NTH1* promoter-*NTH1*^{WT}-reporter-3XFLAG (first 95 aa) was integrated into the *URA3* locus
JE650 (reporter)	*MAT α, cln1Δ, cln2Δ, cln3::leu2, lexOPr-Cln1-Leu2, ADE2, his3::cyc1-Pr-lexO TF-his3, TRP, ura3::URA3 NTH1*^{S66A} *-reporter-3XFLAG*	like above, but *NTH1*^{S60A}-reporter-3XFLAG (first 95 aa)
JE651 (reporter)	*MAT α, cln1Δ, cln2Δ, cln3::leu2, lexOPr-Cln1-Leu2, ADE2, his3::cyc1-Pr-lexO TF-his3, TRP, ura3::URA3 NTH1*^{S20,21,60,83A} *-reporter-3XFLAG*	like above, but *NTH1*^{S20,21,60,83A}-reporter-3XFLAG (first 95 aa)
JE653 (reporter)	*MAT α, cln1Δ, cln2Δ, cln3::leu2, lexOPr-Cln1-Leu2, ADE2, his3::cyc1-Pr-lexO TF-his3, TRP, ura3::URA3 NTH1*^{S60A} *-reporter-3XFLAG*	like above, but *NTH1*^{S60A}-reporter-3XFLAG (first 95 aa)
JE654 (reporter)	*MAT α, cln1Δ, cln2Δ, cln3::leu2, lexOPr-Cln1-Leu2, ADE2, his3::cyc1-Pr-lexO TF-his3, TRP, ura3::URA3 NTH1*^{S83A} *-reporter-3XFLAG*	like above, but *NTH1*^{S83A}-reporter-3XFLAG (first 95 aa)
JE658 (reporter)	*MAT α, cln1Δ, cln2Δ, cln3::leu2, lexOPr-Cln1-Leu2, ADE2, his3::cyc1-Pr-lexO TF-his3, TRP, ura3::URA3 NTH1*^{S20,21A} *-reporter-3XFLAG*	like above, but *NTH1*^{S20,21A}-reporter-3XFLAG (first 95 aa)
JE659 (reporter)	*MAT α, cln1Δ, cln2Δ, cln3::leu2, lexOPr-Cln1-Leu2, ADE2, his3::cyc1-Pr-lexO TF-his3, TRP, ura3::URA3 NTH1*^{S60,83A} *-reporter-3XFLAG*	like above, but *NTH1*^{S60,83A}-reporter-3XFLAG (first 95 aa)
KK003 (reporter)	*MAT α, cln1Δ, cln2Δ, cln3::leu2, lexOPr-Cln1-Leu2, ADE2, his3::cyc1-Pr-lexO TF-his3, TRP, ura3::URA3 NTH1*^{S20,21,60,66,83A} *-reporter-3XFLAG*	like above, but *NTH1*^{S20,21,60,66,83A}-reporter-3XFLAG
LD040 (reporter)	*MAT α, cln1Δ, cln2Δ, cln3::leu2, lexOPr-Cln1-Leu2, ADE2, his3::cyc1-Pr-lexO TF-his3, TRP, ura3::URA3 NTH1*^{S20,21,60,66} *-reporter-3XFLAG*	like above, but *NTH1*^{S20,21,60,66A}-reporter-3XFLAG

LD041 (reporter)	*MAT α, cln1Δ, cln2Δ, cln3::leu2, lexOPr-Cln1-Leu2, ADE2, his3::cyc1-Pr-lexO TF-his3, TRP, ura3::URA3* $NTH1^{S20,21,66,83A}$ *-reporter-3XFLAG*	like above, but $NTH1^{S20,21,66,83A}$-reporter-3XFLAG
LD038 (reporter)	*MAT α, cln1Δ, cln2Δ, cln3::leu2, lexOPr-Cln1-Leu2, ADE2, his3::cyc1-Pr-lexO TF-his3, TRP, ura3::URA3* $NTH1^{S21,60,66,83A}$ *-reporter-3XFLAG*	like above, but $NTH1^{S21,60,66,83A}$-reporter-3XFLAG
LD039 (reporter)	*MAT α, cln1Δ, cln2Δ, cln3::leu2, lexOPr-Cln1-Leu2, ADE2, his3::cyc1-Pr-lexO TF-his3, TRP, ura3::URA3* $NTH1^{S20,60,66,83A}$ *-reporter-3XFLAG*	like above, but $NTH1^{S20,60,66,83A}$-reporter-3XFLAG
JE611c	*MAT α, cln1Δ, cln2Δ, cln3::leu2, lexOPr-Cln1-Leu2, ADE2, his3::cyc1-Pr-lexO TF-his3, TRP, URA*	[2]
JE629	*MAT α, cln1Δ, cln2Δ, cln3::leu2, lexOPr-Cln1-Leu2, ADE2, his3::cyc1-Pr-lexO TF-his3, TRP, URA, pho85::kanMx*	like above, *pho85* was deleted

Supplementary Table S2, related to experimental procedures: Plasmids used in this study.

**name**	**description**	**comment/source**
pJE004	*nth1 gene with a N-terminal 6xHis-tag in pET28*	[2]
pJE006	*nth1 gene S66A with a N-terminal 6xHis-tag in pET28*	[2]
pJE014	*nth1 gene with PKA sites replaced to A S20-21-60-83, with a N-terminal 6xHis-tag in pET28*	$NTH1^{S20,20,60,83A}$ gene was inserted into pET28
pLD006	*nth1 gene L19A with a N-terminal 6xHis-tag in pET28*	$NTH1^{L19A}$ gene was inserted into pET28
	bmh1 gene with N-terminal 6xHis-tag in pET15b	Kind gift from Veronika Obšilová[3] .
pLD013	*nth1 gene with C-terminal Strep-tag in pASK-IBA3*	Nth1 gene was inserted into pASK-IBA3 (IBA, Göttingen, Germany, kind gift from the Karl Forchhammer lab)

Supplementary Table S3, related to figures 2,5,6, and S2,3: Absolute trehalose concentrations.

**Genotype**	**starting OD_{600}**	**Starting concentration of trehalose (nmol extracted from 1.5 ml culture)**
NTH1^{WT}	1.39	260
NTH1^{WT}	1.36	274
NTH1^{WT}	1.10	171
NTH1^{WT}	1.27	186
NTH1$^{\Delta PKA}$	1.40	307
NTH1$^{\Delta PKA}$	1.49	323
NTH1$^{\Delta PKA}$	1.11	228
NTH1^{S20,21A}	1.33	309
NTH1^{S20,21A}	1.41	342
NTH1^{S20,21A}	1.35	203
NTH1^{S20A}	1.24	250
NTH1^{S20A}	1.45	258
NTH1^{S20A}	1.42	212
NTH1^{S21A}	1.18	194
NTH1^{S21A}	1.23	236
NTH1^{S21A}	1.69	235
NTH1^{S60,83A}	1.27	284
NTH1^{S60,83A}	1.41	260
NTH1^{S60,83A}	1.32	285
NTH1^{S60A}	1.60	293
NTH1^{S60A}	1.60	283
NTH1^{S60A}	1.60	310
NTH1^{S83A}	1.70	283
NTH1^{S83A}	1.80	272
NTH1^{S83A}	1.70	277
NTH1^{S66A}	1.44	349
NTH1^{S66A}	1.20	241
NTH1^{S66A}	1.43	306
NTH1^{L19A}	1.38	220
NTH1^{L19A}	1.25	203
NTH1^{L19A}	1.30	185
NTH1^{L19A}	1.62	274
NTH1$^{\Delta 1\text{-}24}$	1.60	271
NTH1$^{\Delta 1\text{-}24}$	1.60	289
NTH1$^{\Delta 1\text{-}24}$	1.60	283
NTH1^{S20,21E}	1.60	309
NTH1^{S20,21E}	1.90	257
NTH1^{S20,21E}	1.80	263

nth1Δ	1.22	263
nth1Δ	1.28	255
nth1Δ	1.19	174
NTH1^{WT} (3 mM GMM)	1.30	236
NTH1^{WT} (3 mM GMM)	1.42	266
NTH1^{WT} (3 mM GMM)	1.37	214
Cell cycle arrest/starvation (JE611c)	1.7	30
Cell cycle arrest/starvation (JE611c)	1.9	28
Cell cycle arrest/starvation *pho85Δ* (JE629)	1.6	41
Cell cycle arrest/starvation *pho85Δ* (JE629)	1.8	41

Supplementary Figures:

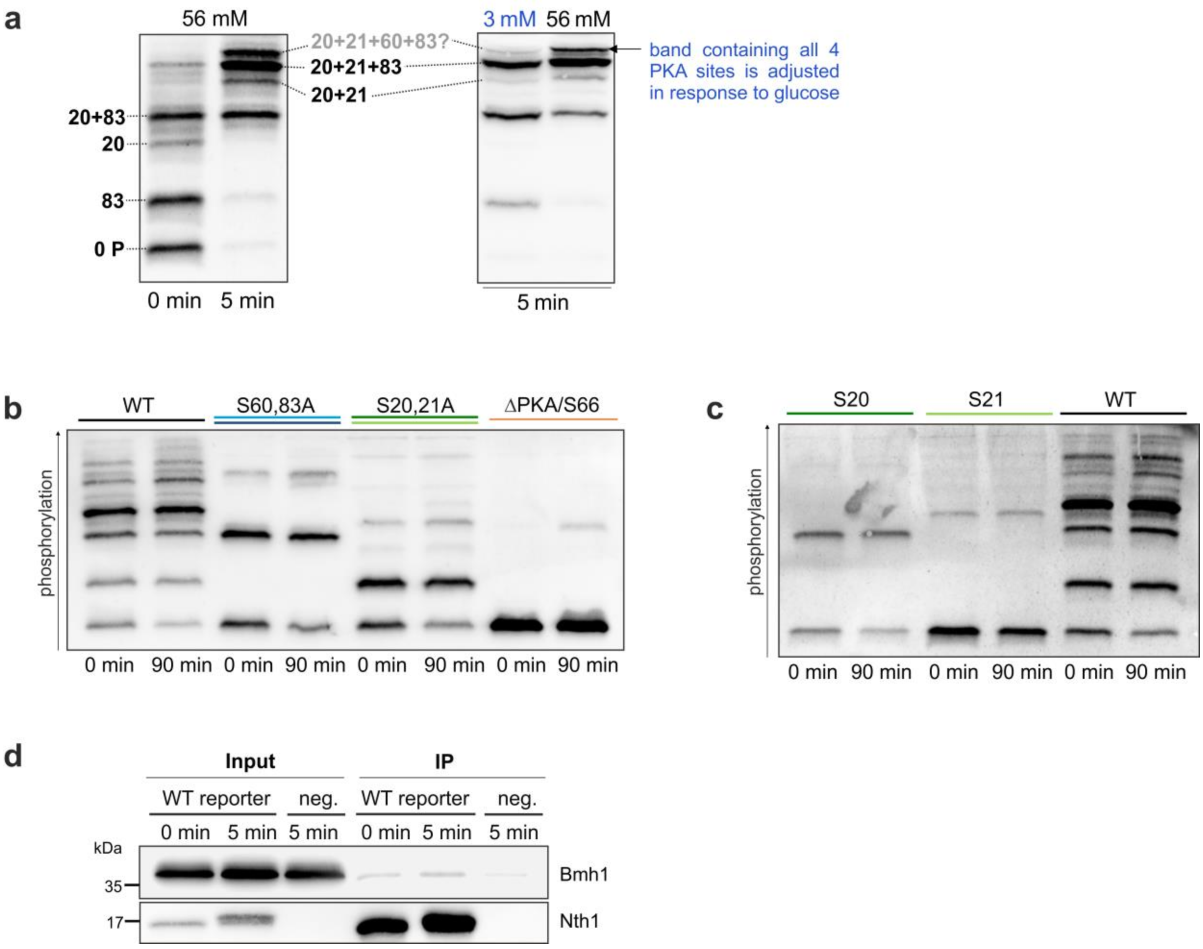

Supplementary Figure S1, related to Figures 2, 3 and 4. Further characterization of the Nth1 reporter construct. (**a**) Summary of the identified phospho-isoforms of Nth1 *in vivo.* These are lanes from the blots shown in main Figure 2d (left) and 2c (right) with annotations based on the mutant analysis in Figures 5,6,7. Also see additional supplementary information below for the uncut gel. Phos-tag-SDS-PAGE-western analysis of the reporter construct of stationary cells and after re-addition of 3 mM or 56 mM glucose is shown. Single and double phosphorylated isoforms of the perfect PKA sites S20 and S83 exist already in the stationary phase with very low PKA activity. After glucose re-addition, phospho-isoforms containing S20+21, S20+21+83, and most likely the combination of all four PKA sites S20,21,60,83 appear. In response to different glucose concentrations, resulting in different PKA activities, the amount of the highest band as the probably only band containing a phospho-isoform with both Bmh1 binding sites S60 and 83 is adjusted. Since both Bmh1 binding sites S60 and 83 are required for activation, the highest band is most likely the active phospho-isoform of Nth1. (**b**) and (**c**) Phos-tag SDS-PAGE of cells expressing 3x-FLAG-tagged Nth1-reporter constructs of (**b**) mutants of the PKA sites and (**c**) single site addbacks in a 5A background integrated into

cln1Δcln2Δcln3Δ LexApr-CLN1. The cells were grown on ethanol minimal medium containing hormone, arrested in G_1, and synchronously released into the cell cycle by addition of hormone. (**d**) Co-Immunoprecipitation of the 3xFLAG-tagged $Nth1^{WT}$-reporter with V5-tagged Bmh1. Cells were grown for 4 days until stationary phase and recovered by dilution in 1 % GMM. In contrast to the full-length protein, the reporter does not efficiently pull down Bmh1.

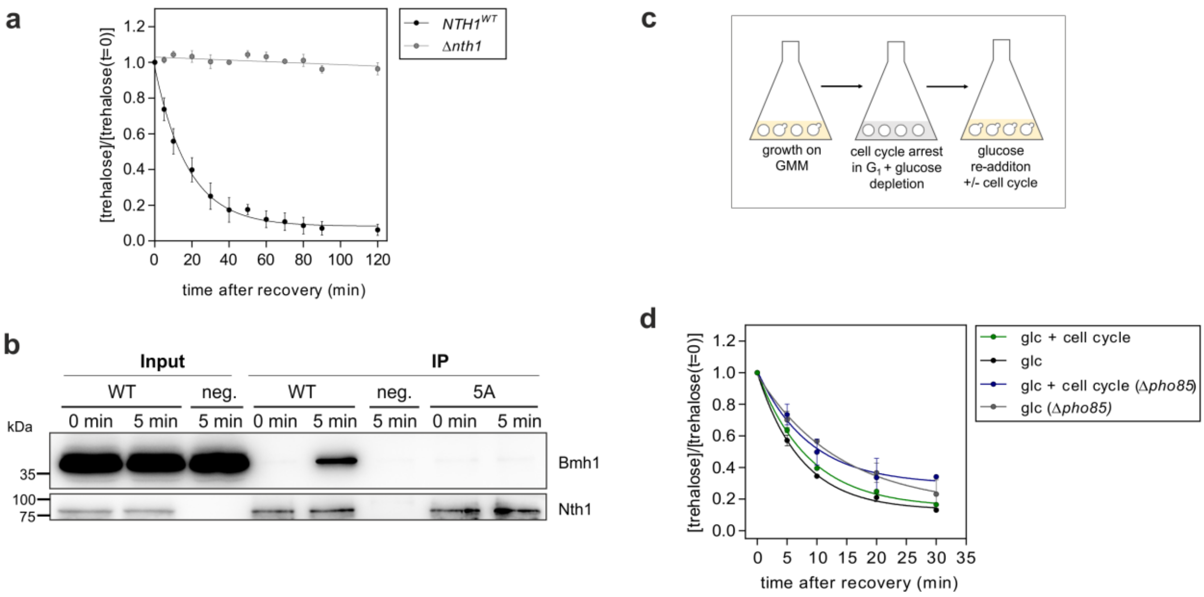

Supplementary Figure S2, related to Figure 5: Control experiments related to Nth1 activity. (**a**) and (**b**) Cells were grown for 4 days until stationary phase and recovered by dilution in 1 % GMM. (**a**) Normalized trehalose concentration after recovery from stationary phase. SEM for at least three biological replicates with two technical replicates each. All trehalose concentrations were normalized to the concentration at t = 0 min. See Supplementary Table S3 for absolute concentrations. (**b**) Co-Immunoprecipitation of full-length 3x-FLAG tagged Nth1 with V5-tagged Bmh1. Cells expressing untagged Nth1 and V5-tagged Bmh1 was used as negative control. (**c**) Experimental schematic: Recovery from G_1 arrest with glucose depletion. WT or Δ*pho85* cells, both in the hormone-inducible strain background, were simultaneously starved and arrested in G_1 for 6 h. Cells were recovered by addition of glucose (1 % final) with or without being released into the cell cycle by addition of hormone. For G_1 arrested cells that are glucose-starved, two sequential 9 h and 15 h pre-cultures were grown on 1 % GMM supplemented with 50 mM potassium phthalate containing 80 nM and 50 nM β-estradiol, respectively. Cells were arrest for 6 h in 0.5 % glucose minimal medium supplemented with 50 mM potassium phthalate (starting OD_{600} was ~1.2). Cells were diluted in glucose minimal medium to a final concentration of 1 % glucose with or without addition of 200 nM β-estradiol. To verify glucose depletion, glucose was measured with a commercial kit from (K-GLUHK, Megazyme). (**d**) Normalized trehalose concentration after recovery from G_1 arrest with glucose depletion as described in (**c**). SEM for two biological replicates with two technical replicates each. All trehalose concentrations were normalized to the concentration at t = 0 min. See Supplementary Table S3 for absolute concentrations.

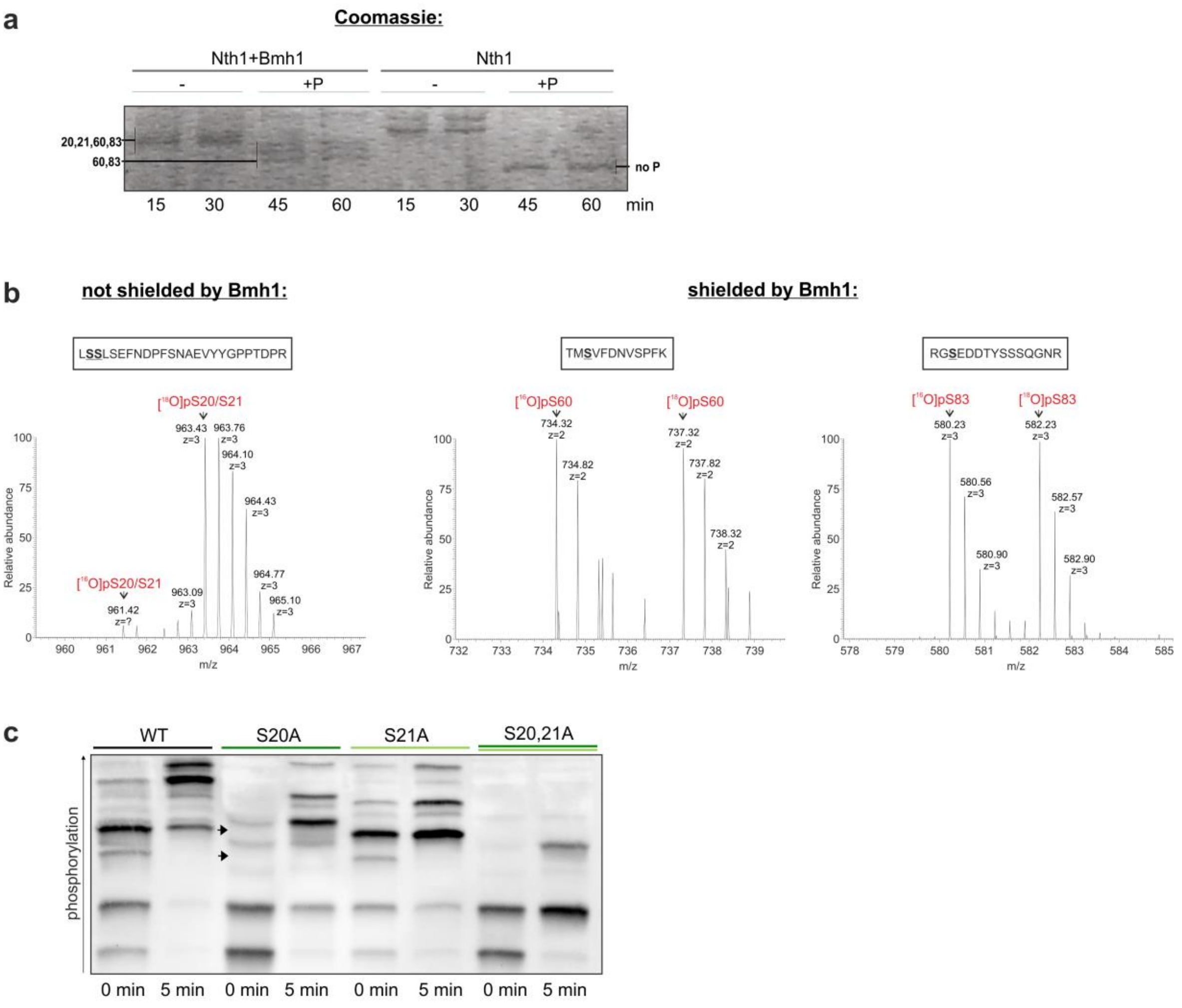

Supplementary Figure S3, related to Figure 6: S20 and S21 are phosphorylated *in vitro* and *in vivo* but not shielded by Bmh1. (**a**) Coomassie of the autoradiograph of Figure 6a. Phos-tag SDS-PAGE of an *in vitro* shielding assay of recombinant 6x-His tagged Bmh1 and full-length 6x-His-tagged Nth1. Nth1 was phosphorylated by PKA in presence or absence of Bmh1 (5:1, Bmh1:Nth1). At the 30 min time point PKA inhibitor and lambda phosphatase were added. (**b**) Mass spectrometry analysis of an *in vitro* shielding assay of Nth1 and Bmh1 as shown on the left panel in Figure 6a. PKA phosphorylation was performed with normal isotopic ATP [(16O)-ATP] or heavy ATP [(18O)-ATP] in the presence of Bmh1. Sample phosphorylated with (16O)-ATP was treated with lambda phosphatase. Mass spectrometry analysis was performed as described previously[4]. (**c**) Phos-tag SDS-PAGE of cells expressing 3x-FLAG-tagged Nth1-reporter constructs. Cells were grown for 4 days until stationary phase and recovered by dilution in 1 % GMM.

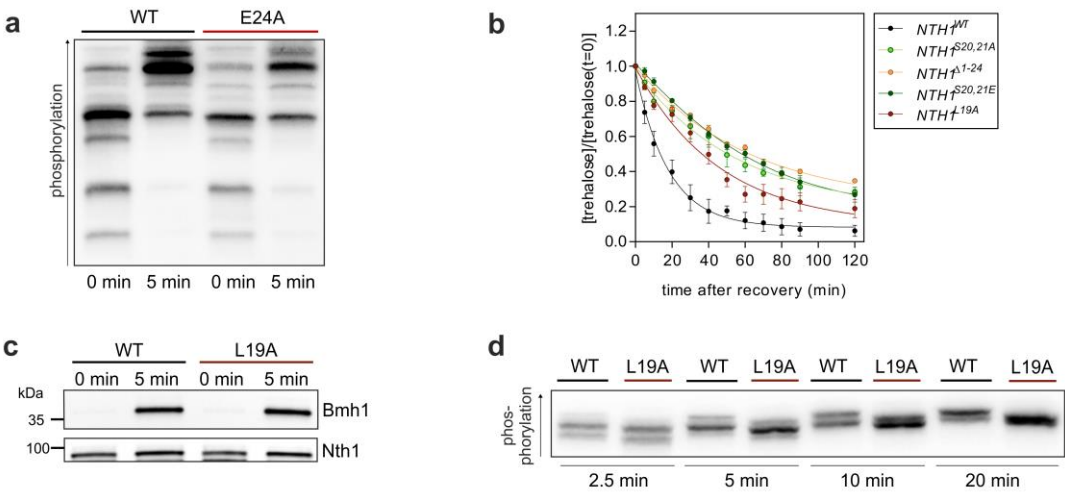

Supplementary Figure S4, related to Figure 7: Further characterization of the N-terminal region surrounding S20 and S21. (**a**) - (**c**) Cells were grown for 4 days until stationary phase and recovered by dilution in 1 % GMM. (**a**) Phos-tag SDS-PAGE of cells expressing 3x-FLAG-tagged Nth1-reporter constructs. (**b**) Normalized trehalose concentration after recovery. SEM for at least three biological replicates with two technical replicates each. All trehalose concentrations were normalized to the concentration at t = 0 min. See Supplementary Table S3 for absolute concentrations. (**c**) Co-Immunoprecipitation of cells expressing full-length 3x-FLAG tagged Nth1 and V5-tagged Bmh1. (**d**) Autoradiogram of an *in vitro* phosphorylation of recombinant full-length 6x-His-tagged Nth1 with PKA. Reactions were stopped at the indicated time points. Phosphorylated species were separated on Phos-tag-SDS-PAGE.

Supplementary References:

1. Ottoz, D. S., Rudolf, F. & Stelling, J. Inducible, tightly regulated and growth condition-independent transcription factor in Saccharomyces cerevisiae. *Nucleic Acids Res* **42**, e130, doi:10.1093/nar/gku616 (2014).
2. Ewald, J. C., Kuehne, A., Zamboni, N. & Skotheim, J. M. The Yeast Cyclin-Dependent Kinase Routes Carbon Fluxes to Fuel Cell Cycle Progression. *Mol Cell* **62**, 532-545, doi:10.1016/j.molcel.2016.02.017 (2016).
3. Veisova, D. *et al.* The C-terminal segment of yeast BMH proteins exhibits different structure compared to other 14-3-3 protein isoforms. *Biochemistry* **49**, 3853-3861, doi:10.1021/bi100273k (2010).
4. Kõivomägi, M. *et al.* Multisite phosphorylation networks as signal processors for Cdk1. *Nat Struct Mol Biol* **20**, 1415-1424, doi:10.1038/nsmb.2706 (2013).

Additional Supplementary Information:

In favor of maximal transparency, we here include all gels and plots which are presented in the manuscript as indicated in full length and uncut.

Figure 1

a

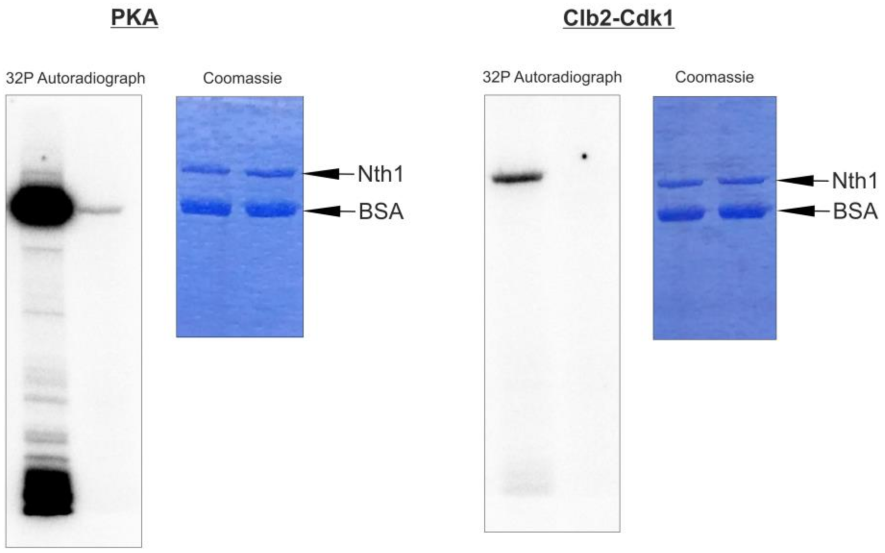

c

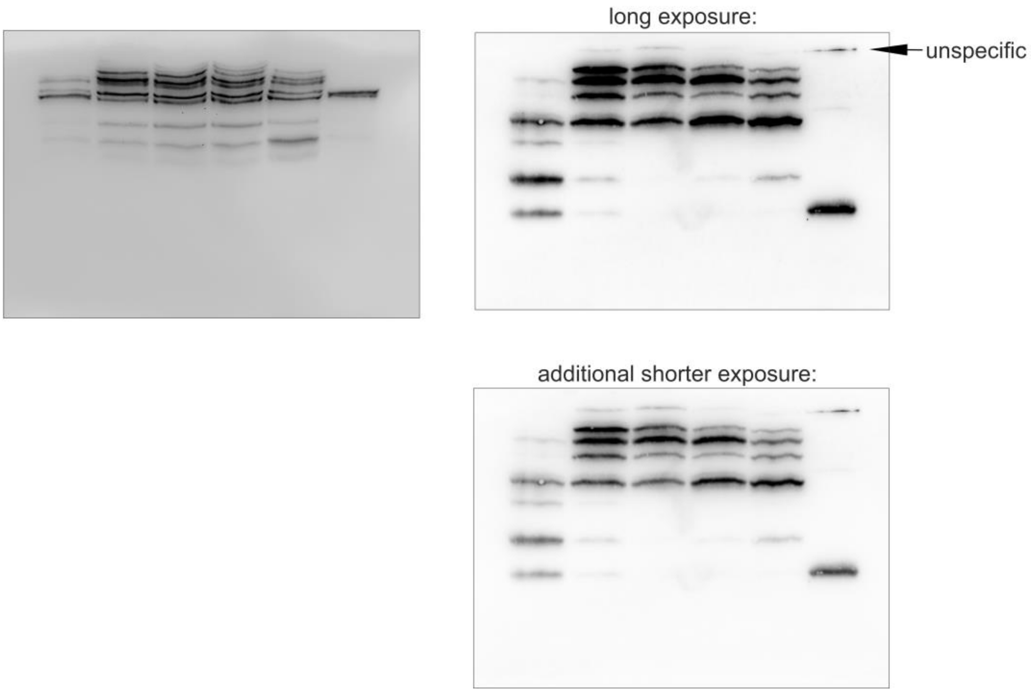

Figure 2

c

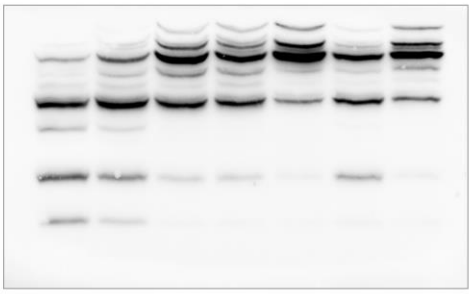

d

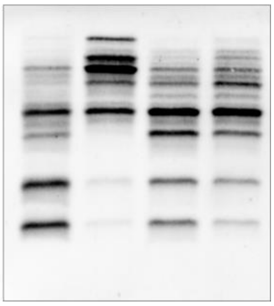

Figure 3

a

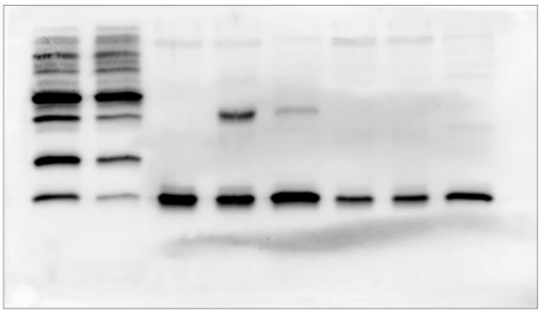

b

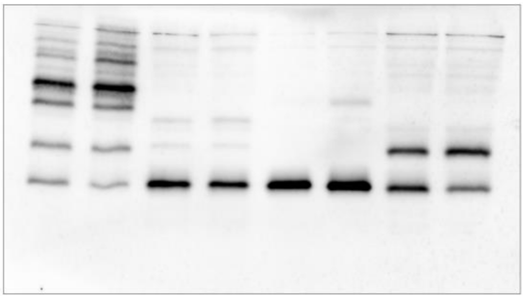

c

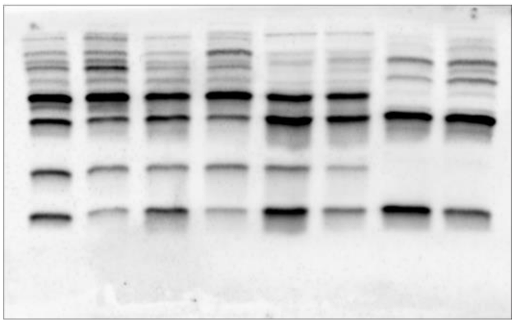

Figure 4

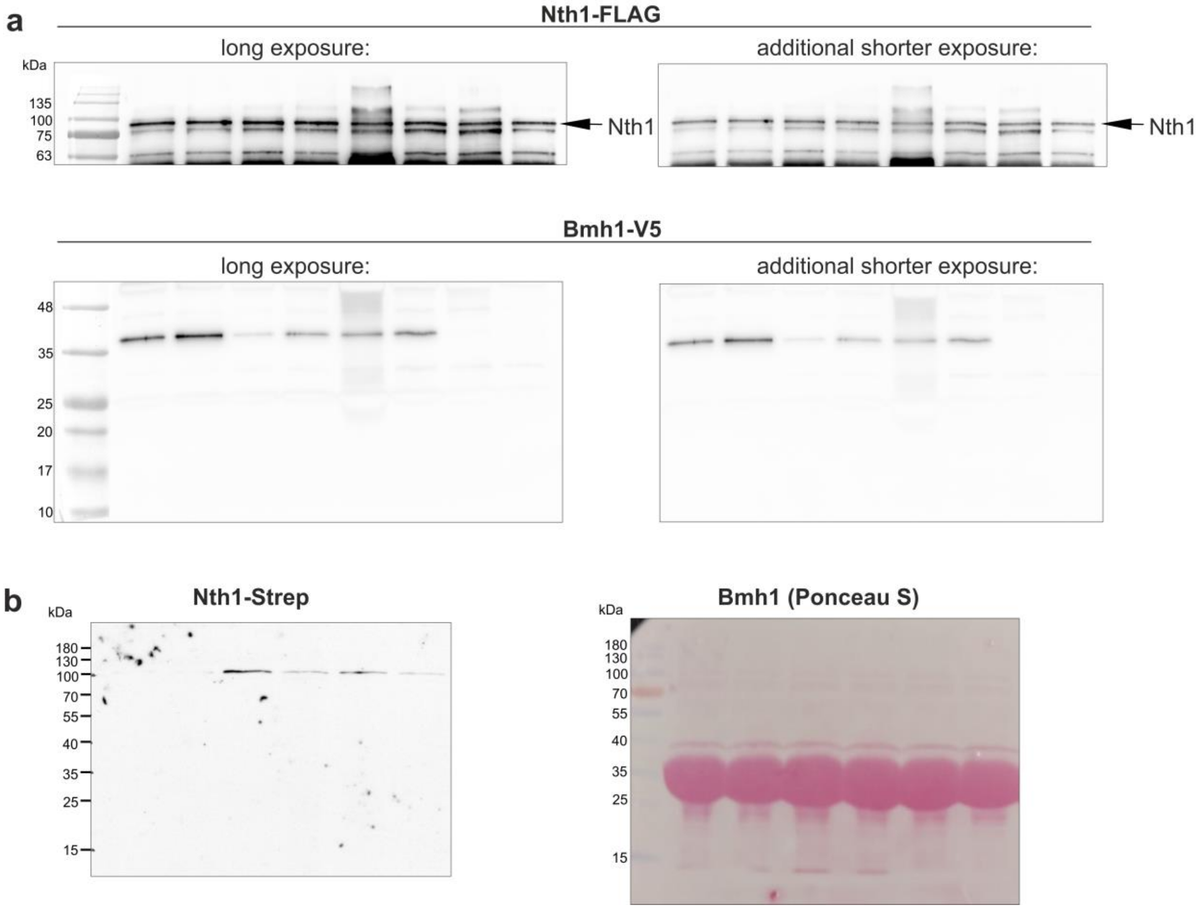
a
Nth1-FLAG
long exposure:
additional shorter exposure:
kDa
135
100
75
63
Nth1
Nth1
Bmh1-V5
long exposure:
additional shorter exposure:
48
35
25
20
17
10
b
Nth1-Strep
kDa
180
130
100
70
55
40
35
25
15
Bmh1 (Ponceau S)
kDa
180
130
100
70
55
40
35
25
15

Figure 5

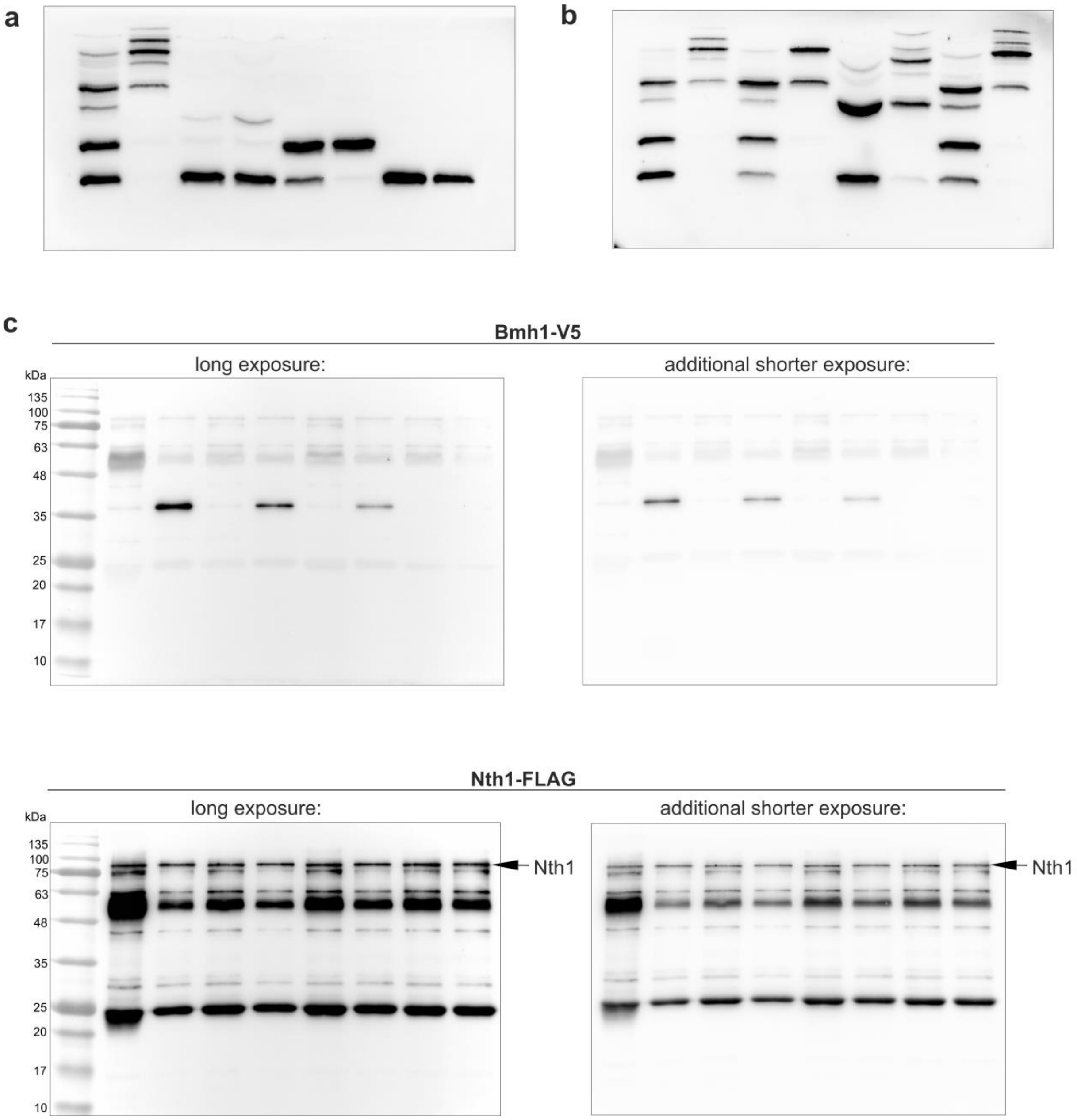

a
b
c
Bmh1-V5
long exposure:
additional shorter exposure:
kDa
135
100
75
63
48
35
25
20
17
10
Nth1-FLAG
long exposure:
additional shorter exposure:
kDa
135
100
75
63
48
35
25
20
17
10
Nth1
Nth1

Figure 6

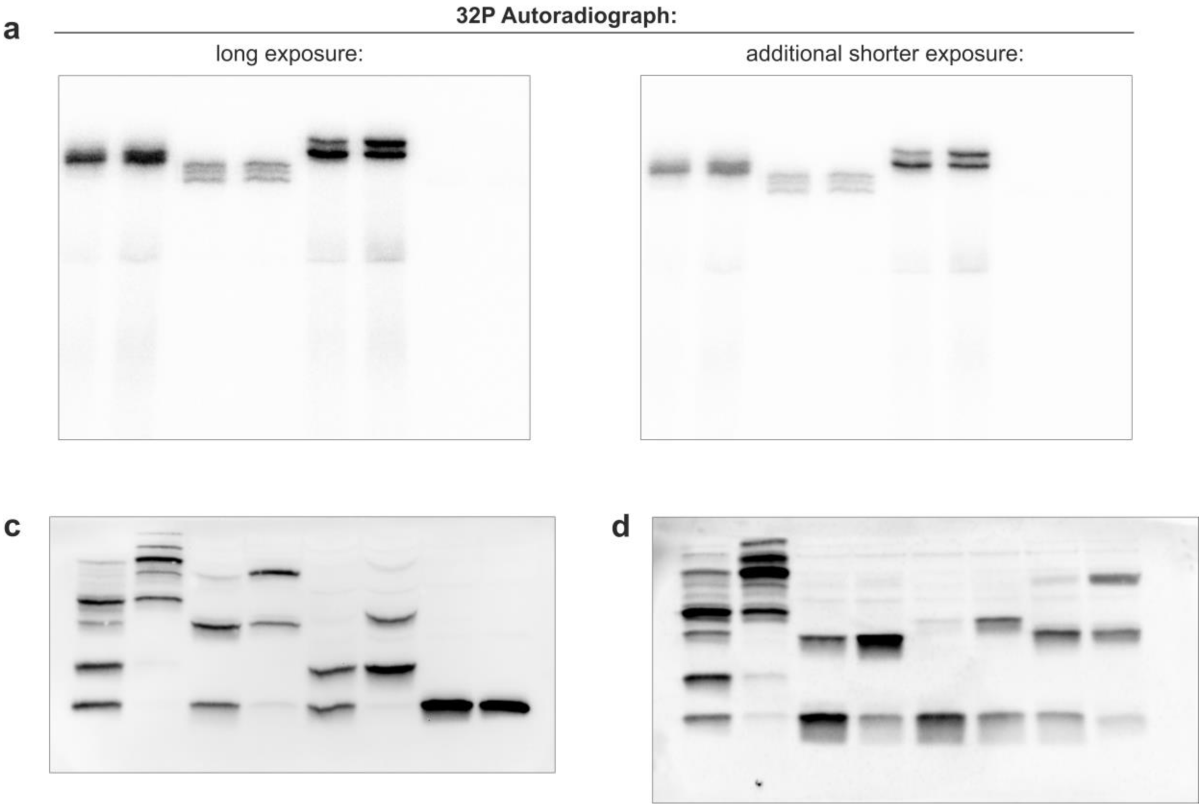

Figure 7

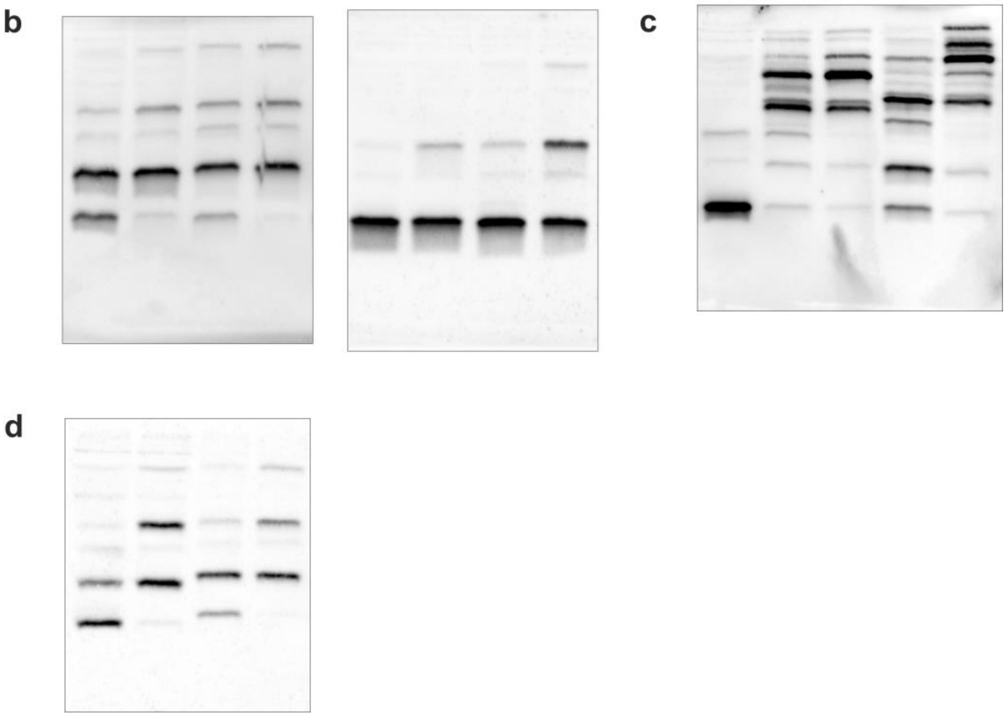

Supplementary Figure S1

a

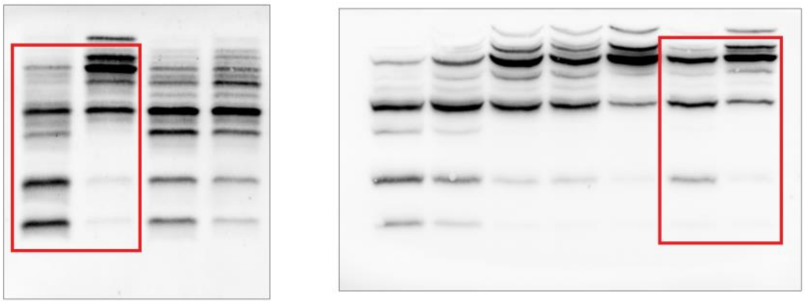

b

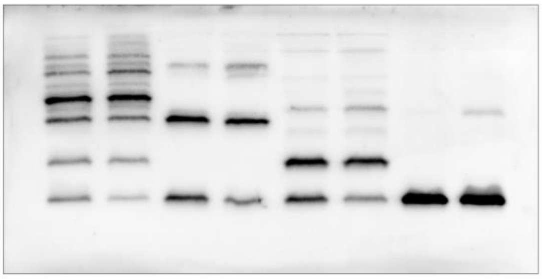

c

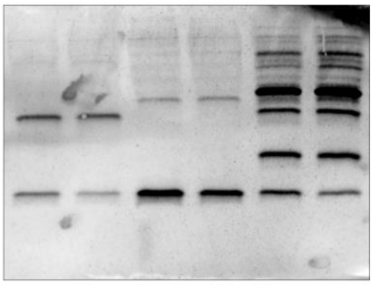

d

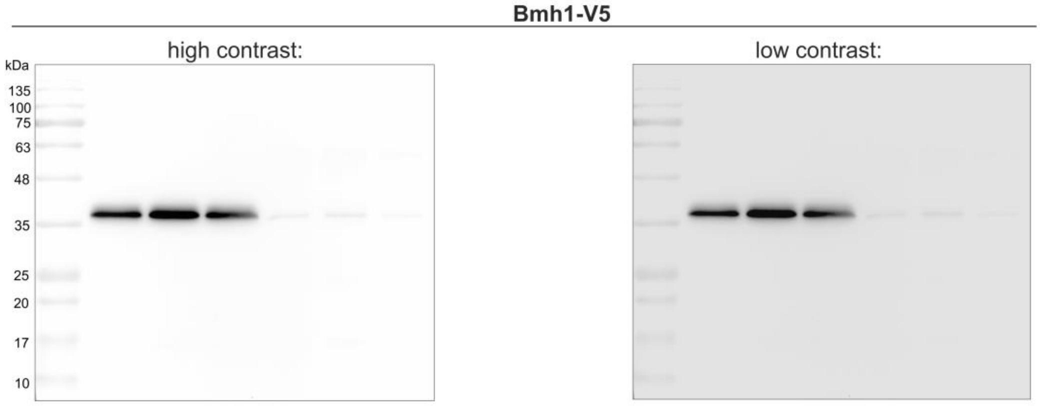

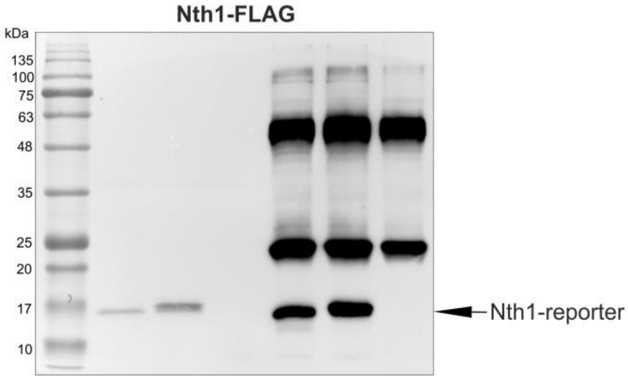

Supplementary Figure S2

b

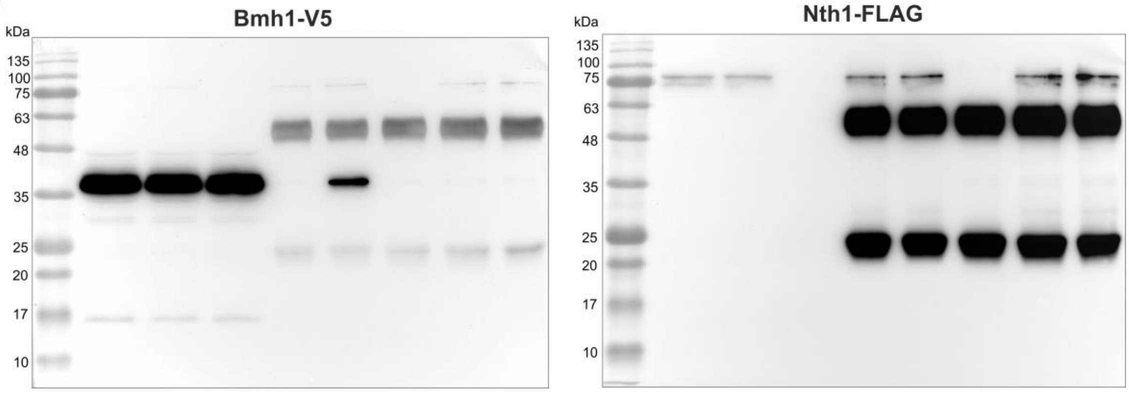

Supplementary Figure S3

a

Coomassie

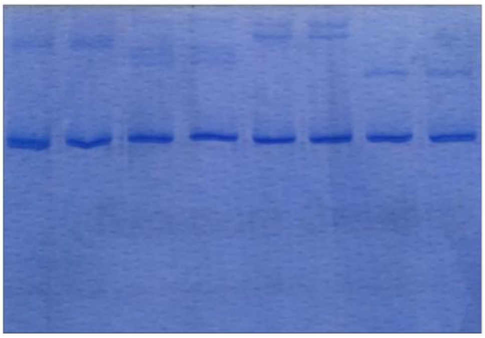

c

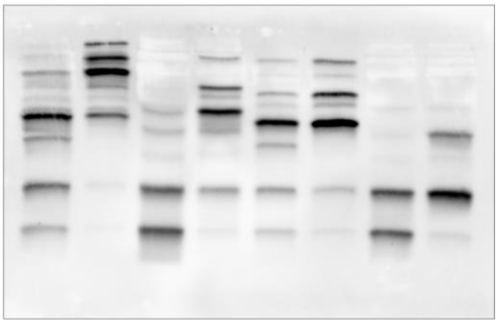

Supplementary Figure S4

a

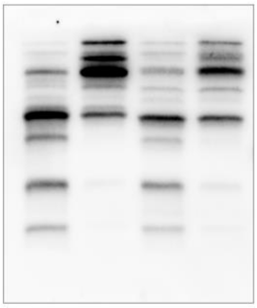

c

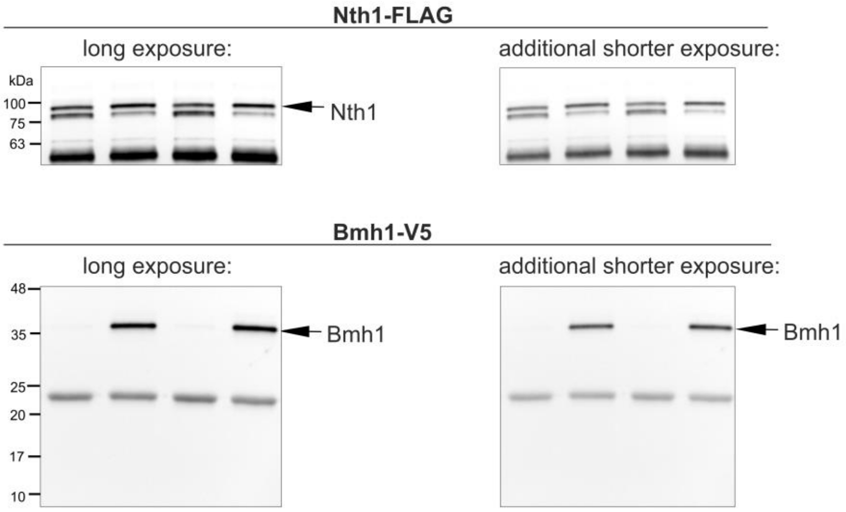

d

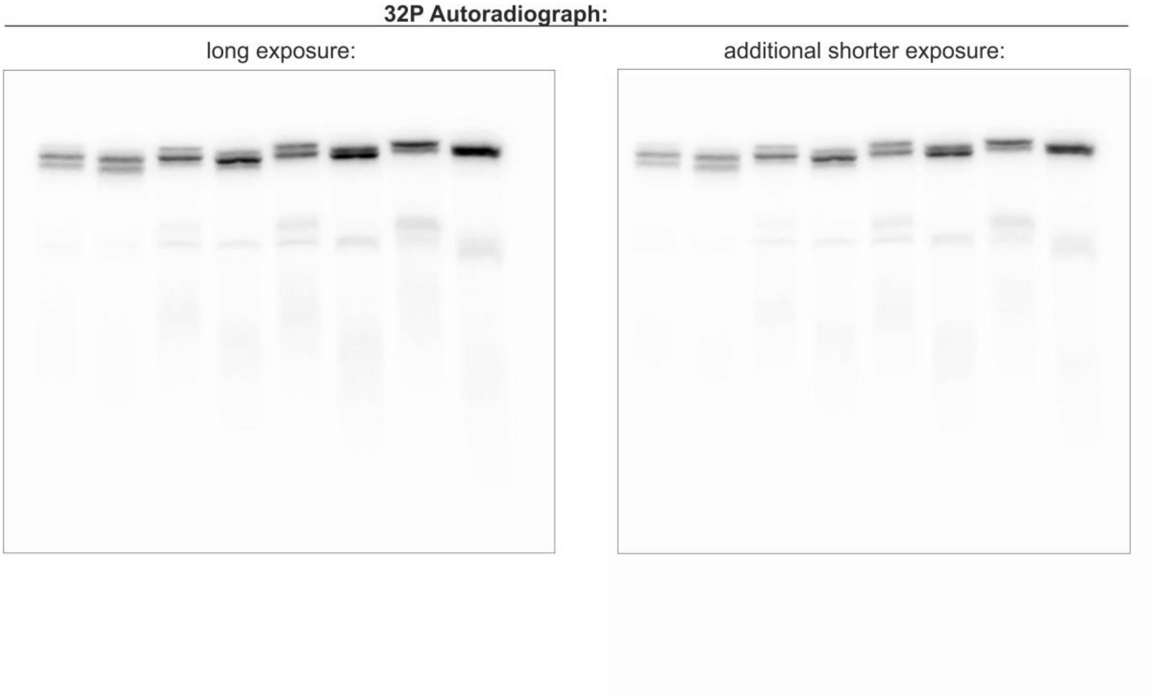

Supplement: Supplementary file 1 — Supplementary Information. [file 41598_2020_80357_MOESM1_ESM.pdf]
